# Angle’s Class II division 1 associated to mandibular retrusion and skeletal open bite: a 5-year post-orthodontic/orthopedic treatment follow-up

**DOI:** 10.1590/2177-6709.22.5.098-112.bbo

**Published:** 2017

**Authors:** Gustavo Tirado Rodrigues

**Affiliations:** 1 Universidade Tiradentes, Curso de Especialização em Ortodontia (Aracaju/SE, Brazil).

**Keywords:** Angle’s Class II Malocclusion, Mandible Retrusion, Open bite, Corrective Orthodontics, Stability

## Abstract

Obtaining long term stability allied to functional and aesthetic balance is the main goal of any orthodontic-orthopedic therapy. This case report describes the orthodontic therapy applied to a 7-year-9-month old child, who presented a Class II, division 1 malocclusion associated to skeletal open bite. Functional and skeletal corrections (sagittally and vertically) were obtained by means of mandible advancement achieved with a closed Balter’s bionator appliance followed by a fixed appliance. This approach showed to be efficient in accomplishing both functional and aesthetic goals, that were kept stable five years after the treatment was finished. This case report was presented to the Board of Directors of the Brazilian Board of Orthodontics and Facial Orthopedics (BBO), as partial requirement to becoming a Diplomate of the BBO.

## RESUMO

A obtenção do equilíbrio funcional e estético em um contexto de estabilidade em longo prazo é o objetivo de qualquer abordagem ortodôntico-ortopédica. O presente relato aborda o tratamento ortodôntico realizado em uma criança com 7 anos e 9 meses de idade, portadora de Classe II, divisão 1, associada a mordida aberta esquelética. A correção funcional e esquelética (sagital e vertical) foi obtida por meio de avanço mandibular, com aparelho Bionator de Balters do tipo fechado, seguido de aparelho ortodôntico fixo. Essa abordagem foi eficiente para atingir as metas funcionais e estéticas, que continuaram estáveis cinco anos após a conclusão do tratamento. Esse caso clínico foi apresentado à Diretoria do Board Brasileiro de Ortodontia e Ortopedia Facial (BBO), como parte dos requisitos para a obtenção do título de Diplomado pelo BBO.

## INTRODUCTION

This case report describes the orthodontic treatment of a 7-year-9-month old male patient, during the second transitional period of mixed dentition, who presented at the clinic for treatment with the chief complaint of having excessively protruded teeth (“too flared”). According to the mother, a slight advancement had been accomplished by the previous orthodontic intervention, during which a fixed palatal bar was used. After an otolanryngologic assessment, the boy was diagnosed as a partial mouth breather and presented a diffuse nasal edema, with hypertrophic turbinates and adenoids. Despite those findings, the case was treated non-surgically. The clinical examination revealed satisfactory hygiene and a low cavity rate. Primary canines (53, 63 and 83) were prematurely lost.

## DIAGNOSIS 

**Figure 1:**
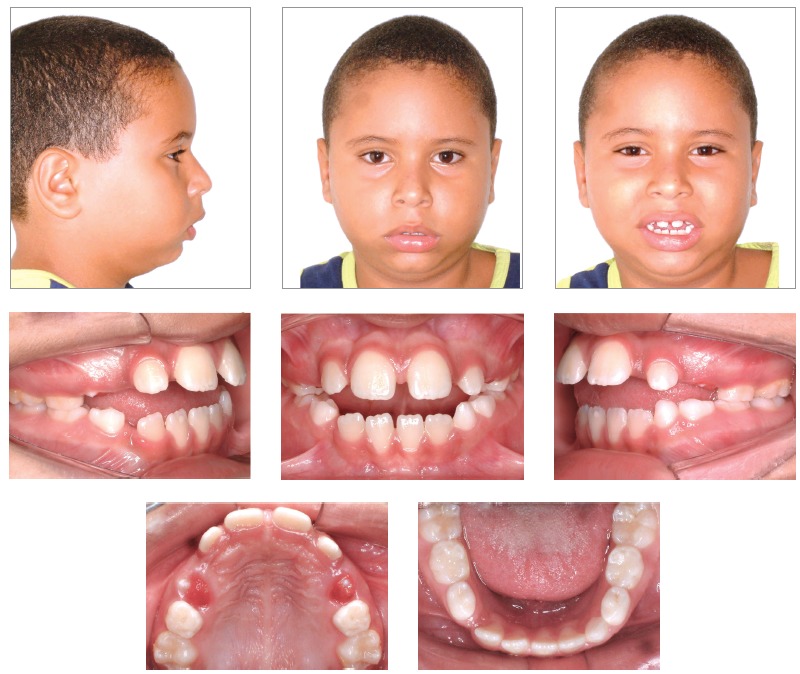
Intraoral and facial initial pictures.

Patient’s face presented marked features of chronic mouth-breathing, associated to a severe muscle hypotonia and an everted lower lip. A light protrusive functional deviation was observed during mandible closure, together with speech impairment, anteriorly positioned tongue and thumb sucking habit. Patient also lacked passive lip closure (5.0 mm), with a rather hypotonic upper lip. His lower lip was both hypotonic and everted. Although the profile was quite convex, given the severe mandible deficiency, nasolabial angle was normal and smile line was tending from normal to low (Fig 1).

**Figure 2:**
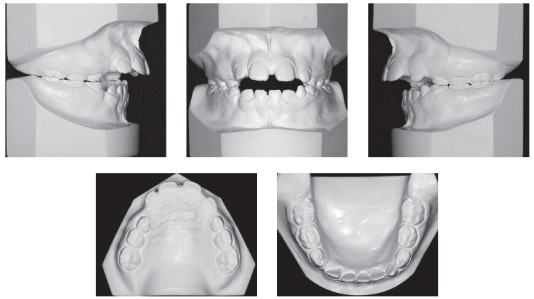
Initial models.

Intraoral assessment (Fig 1, 2) revealed a Class II division 1 malocclusion, with 9.7 mm overjet, anterior open bite and a 6.0 mm negative overbite between upper and lower central incisors. Lower midline presented a 2.5 mm shift to the right due to the premature loss of element #83, jeopardizing the space for the permanent successor (#43). During Class I model manipulation, a 5.5 mm cross-sectional maxillary deficiency was observed between first primary molars and the opposing teeth. 

**Figure 3:**
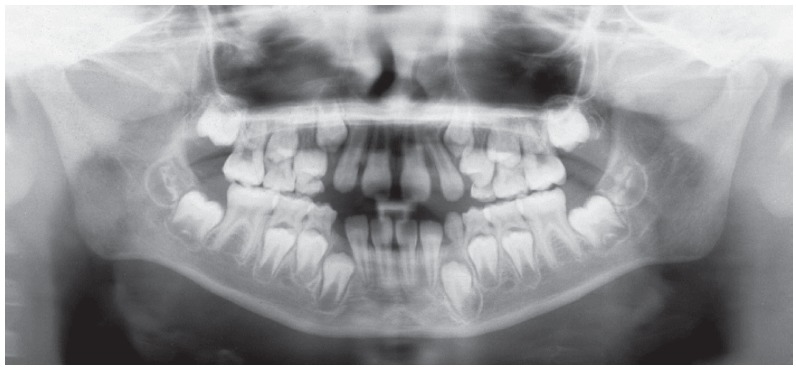
Initial panoramic radiograph.

Initial panoramic radiograph (Fig 3) revealed additional eruption difficulties related to permanent upper canines, imposed by the severe anterior diastemas, that by far exceeded the typical “ugly duckling” phase^1^. It was also verified the presence of all permanent teeth, except for the upper third molars, still under development, as expected for that age group. Root contour, periodontal ligament space and bony crests showed no particular findings and presented quite normal. 

**Figure 4:**
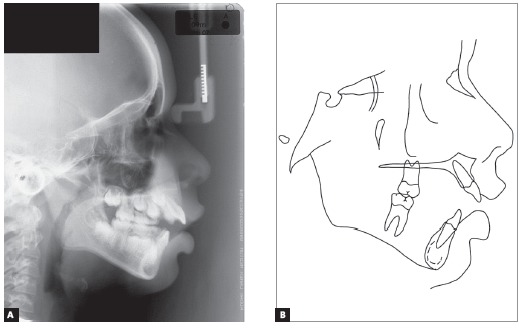
Initial profile cephalometric radiograph (A) and cephalometric tracing (B).

Cephalometric assessment (Fig 4) and values measured throughout the treatment (Table 1) revealed a severe Class II skeletal pattern (ANB = 9.5^o^, Wits = 4.5 mm), given the mandible retrusion (SNA = 83^o^, SNB = 73.5^o^, Facial angle = 82^o^). The clockwise rotation tendency observed in patient’s profile through both SNGoGn (35^o^) and Axis Y (61^o^) would limit skeletal Class II and open bite corrections. There was a strong genetic factor associated to the Class II, as his father’s profile was also seen to be severely convex from the skeletal perspective (Convexity angle = 22^o^). Upper and lower incisors were proclined (Interincisal angle = 116^o^) and with increased axial inclination (1.NA = 26^o^; 1. NB = 30^o^, IMPA=99^o^).

**Table 1:** Cephalometric values: A) Initial, A1) intermediate, B) final and C) 5 years post containment.

	Measurements		Normal	A	A_1_	B	C	Dif. A/B
Skeletal pattern	SNA	(Steiner)	82^o^	83°	82°	77.5°	77.5°	5.5°
	SNB	(Steiner)	80^o^	73.5°	74.5°	71°	73°	1.5°
	ANB	(Steiner)	2^o^	9.5°	7.5°	6.5°	4.5°	3°
	Wits	(Jacobson)	♀ 0 ±2 mm ♂ 1 ±2 mm	4.5 mm	2 mm	3.5 mm	2.5 mm	1 mm
	Angle of convexity	(Downs)	0^o^	22°	19°	17°	12°	5°
	Y-axis	(Downs)	59^o^	61°	61°	60°	61°	1°
	Facial angle	(Downs)	87^o^	82°	83°	84°	84°	-2º
	SN-GoGn	(Steiner)	32^o^	35°	36°	40°	37°	-5°
	FMA	(Tweed)	25^o^	26°	27.5°	27°	25°	-1°
Dental pattern	IMPA	(Tweed)	90^o^	99°	95°	96°	99°	3°
	1.NA (degrees)	(Steiner)	22^o^	26°	13°	15°	20°	-11°
	1-NA (mm)	(Steiner)	4 mm	3.5 mm	2 mm	4 mm	7 mm	-0.5 mm
	1.NB (degrees)	(Steiner)	25^o^	30°	27°	29°	31°	-1°
	1-NB (mm)	(Steiner)	4 mm	4.5 mm	7 mm	9.5 mm	10 mm	-5 mm
	- Interincisal angle	(Downs)	130^o^	116°	132°	129°	125°	-13°
	1-APo	(Ricketts)	1 mm	0.5	3	5	7	-4.5
Profile	Upper lip - S-line	(Steiner)	0 mm	6 mm	5 mm	6 mm	5 mm	-
	Lower lip - S-line	(Steiner)	0 mm	5.5 mm	6 mm	6 mm	5 mm	-0.5 mm

Masticatory muscles and temporomandibular joints were asymptomatic to palpation and movement. 

## TREATMENT PLANNING

Due to the significant facial, skeletal and functional involvements, the planning was traced as follows: 1) maxillary cross-sectional approach, with modified Haas appliance, aiming at allowing for future mandible advancement without inducing posterior crossbite; 2) fixed appliance for upper incisors (#12 and #22 in counter-angle position), as to manage the inter-incisors diastemas, facilitating upper canines eruption; and 3) after expander removal, closed Balter’s bionator appliance for mandible advancement, with constructive bite, reducing the overjet by half, followed by a second appliance, posteriorly placed, in order to complete the correction. 

The corrective orthodontic phase was planned with full fixed appliances in both arches (MBT, 0.022 x 0.028-in) and in order to solve the space discrepancy on the lower arch, pre-molars and canines interproximal stripping was planned, under lingual arch anchorage, welded to first molar bands (#36 and #46)^2^. Aligning and levelling were planned with 0.014-in and 0.016-in NiTi archwires, followed by 0.016-in, 0.018-in and 0.020-in stainless steel (SS) archwires, and 0.019 x 0.025-in SS finishing arches, with ideal shape, torque and coordination. During finishing, anterior vertical elastics could be indicated, if necessary, in order to overcorrect the overbite. 

For the retention phase, an upper wraparound-like removable appliance, with palatal grid, for full time use, except during meals, was planned for the first 12 months, coupled with the lower fixed retainers (0.036-in stainless steel) bonded to canines. 

Lingual function and posture assessments were requested from a speech therapist, as well as diagnosis and treatment of the mouth-breathing condition. 

The success of this planning would rely, besides to patient’s cooperation, on the cessation of the negative mouth habit (thumb sucking) as much as on his growth response. Nasal obstruction clinical therapy success and efficiency were key to both the growth response and to the compliance towards the closed Bionator therapy. The vertical growth tendency and the family component to the mandible retrusion would render the orthopedic response slightly less predictable. 

## TREATMENT PROGRESS

As estimated, the maxillary expansion promoted a transient increase of the open bite. On the other hand, it not only created space to accommodate both upper canines into the arch line but also prepared the maxilla for the incoming mandible advancement. The expander appliance itself was kept as a retainer for a period of four months, and removal was only promoted once the palatal suture was proven to be fully ossified. 

Upper anterior diastemas were managed by means of reciprocal forces, with the caveat to maintain the lateral incisors with the roots slightly inclined to the mesial, what allowed for the canines to freely erupt over the alveolar ridge crest, without menacing the roots of the lateral incisors. 

**Figure 5:**
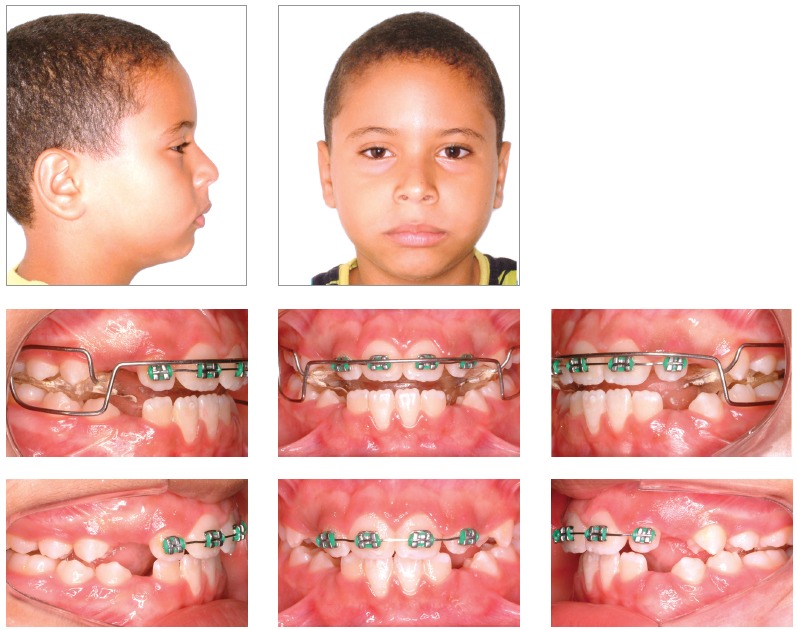
Mandible advancement with closed Balters bionator: facial and occlusal aspects. Improvement on vertical and sagittal aspects.

**Figure 6:**
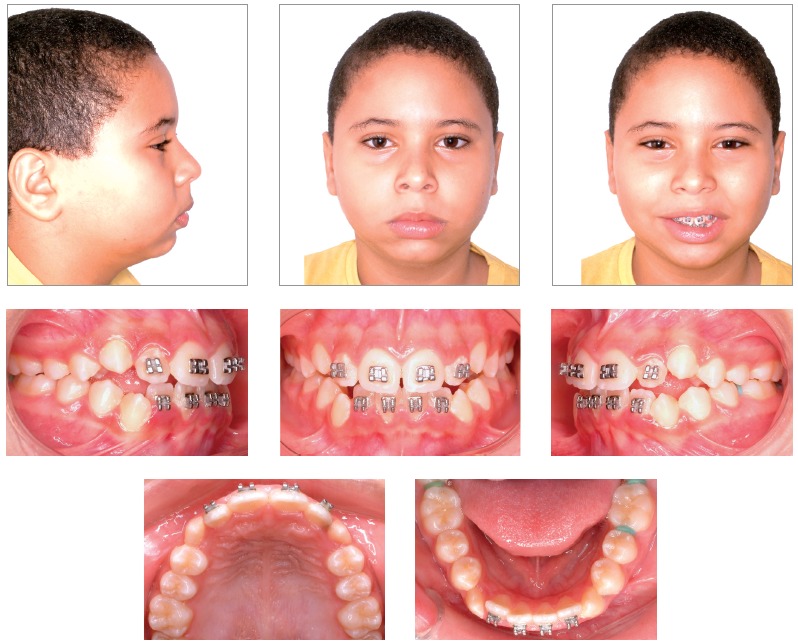
Intermediate intraoral and facial photographs.

**Figure 7:**
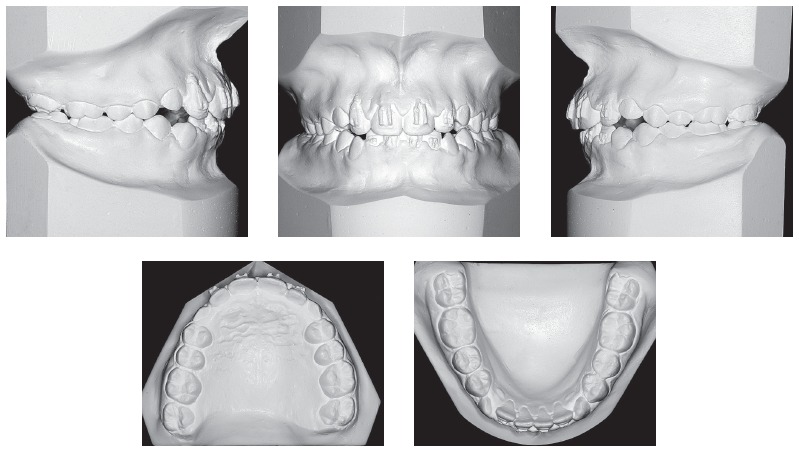
Intermediate models.

**Figure 8:**
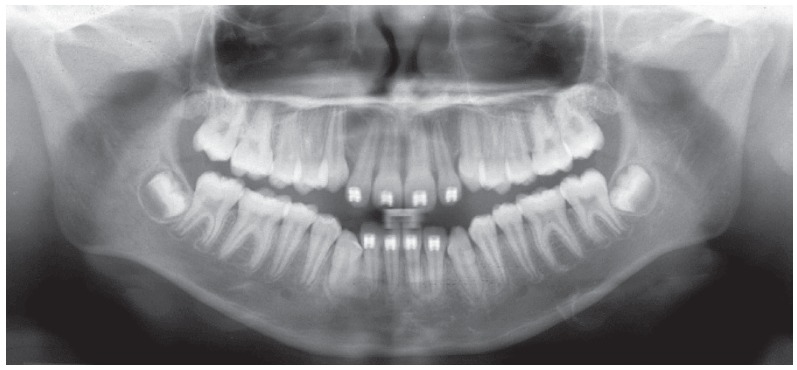
Intermediate panoramic radiograph.

**Figure 9:**
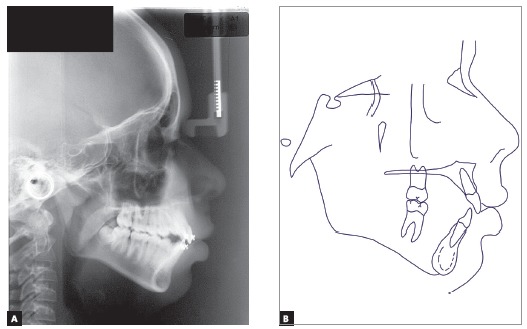
Intermediate profile cephalometric radiograph (A) and cephalometric tracing (B).

The mandible advancement with Balters bionator was necessary in two circumstances in order to make the process more gradual, optimizing adaptive conditions and treatment response.^3^ ^,^ ^4^ The wraparound acrylic splint was key towards correcting the tongue posture and the open bite.^5^ Patient cooperation was rather favorable. After wearing the first appliance for 6 months, without any grinding of the acrylic body at the molar region, a second appliance was cast for the final advancement and fit in an edge-to-edge incisors position (Fig 5). Vertical response required the patient to cease the thumb sucking habit, that was also incompatible with wearing the appliance. At the end of 18 months with the Balters bionator^6^, facial improvements started to be observed, together with changes in both sagittal and vertical aspects of the occlusion, leaving only the interdental stripping space adjustment to the corrective phase. When the interceptive phase was finished, patient was reassessed and a new set of orthodontic records was requested (Figs 6 to 9 and Tab 1). The finishing of the corrective phase required customized wire bending as to achieve the best root parallelism, aesthetic and functional adjustments. The corrective phase lasted one year and five months, and elapsed without any intercurrences.

Retainers were used according to prescription and the speech therapist reassessment did not reveal the need for treatment, since tongue and perioral muscles had recovered normal status. After new radiographs and impressions had been taken, patient was requested to extract lower third molars. Once the upper retainer started to be used only overnight, the opening of a slight interincisal diastema demanded the bonding of a fixed 0.016-in SS retention wire to the palatal aspect of anterior teeth.

## RESULTS 

**Figure 10:**
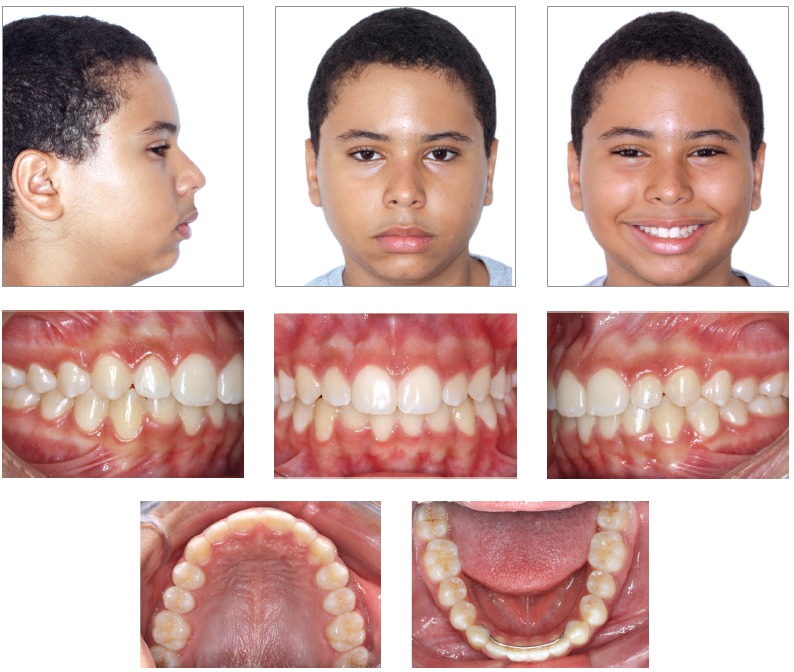
Final intraoral and facial photographs.

**Figure 11:**
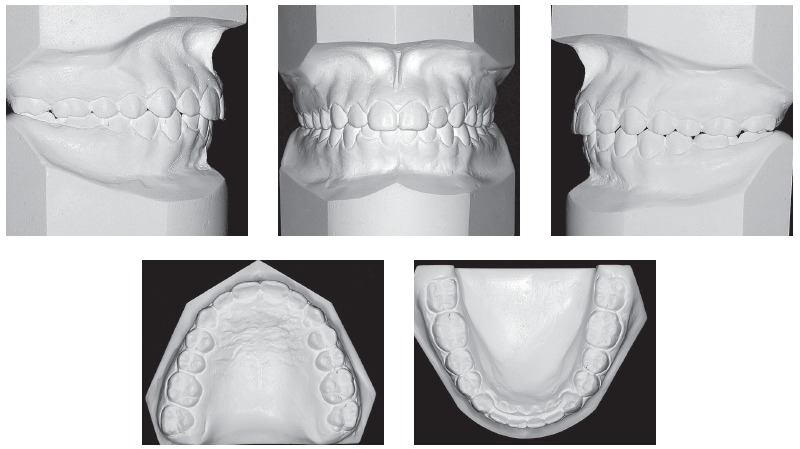
Final models.

The orthodontic-orthopedic approach, allied to patient’s cooperation and good response to treatment, has allowed for a better and less concave facial profile. Passive lip closure was re-established, with a marked improvement of the lower lip position. Profile, however, was still kept somewhat concave, consistent with patient’s ethnical heritage. Smile line improved and allowed for a better exposure of upper teeth. Canine and molar excursion guides as well as improved overbite and overjet were visibly achieved (Figs 10 and 11).

The assessment of the skeletal condition can be seen in Figure 13 and Table 1. There has been a good sagittal response (ANB from 9.5^o^ to 6.5^o^, and Wits from 4.5 mm to 3.5 mm) and improvements on the convexity of the skeletal profile (convexity angle from 22^o^ to 17^o^). However, the skeletal changes primarily impacted the skeletal control of the maxilla (SNA from 83^o^ to 77.5^o^). Mandible also exhibited changes, but only noticeable when assessing the facial angle (from 82^o^ to 84^o^). The discrete changes observed at the mandible were probably caused by the unfavorable clockwise rotation tendency (FMA from 26^o^ to 27^o^ and SNGoGn from 35^o^ to 40^o^).

**Figure 12:**
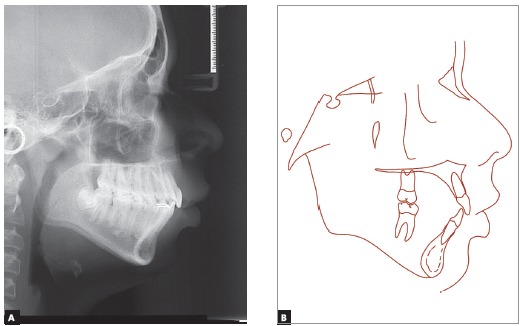
Final profile cephalometric radiograph (A) and cephalometric tracing (B).

**Figure 13:**
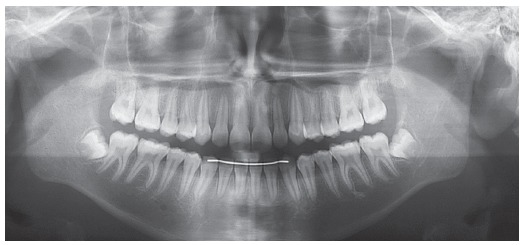
Final panoramic radiograph.

Upper incisors retraction resulting from the use of the orthopedic appliance was kept during the corrective phase (1.NA = 26^o^/ 13^o^/ 15^o^, 1-NA = 3.5/ 2/ 4 mm). Lower incisors were also retracted during the orthopedic phase, despite having suffered a slight protrusion during the orthodontic phase (IMPA = 99^o^/ 95^o^/ 96^o^, and 1.NB = 30^o^/ 27^o^/ 29^o^). As a consequence, interincisal angle was tending towards normality (116^o^/ 132^o^/ 129^o^) (Tab. 1).

Mandible excursion guides were obtained for anterior movements and both left and right lateral movements. Neuromuscular balance was achieved including lip and tongue positions, as well as during swallowing and speaking. Besides that, periodontal and TMJ health were preserved. 

The final panoramic radiograph revealed nice contouring of the roots and good parallelism, together with the alveolar bone crest heights, that were equally preserved (Fig 13).

## FIVE YEAR FOLLOW-UP AFTER TREATMENT

**Figure 14:**
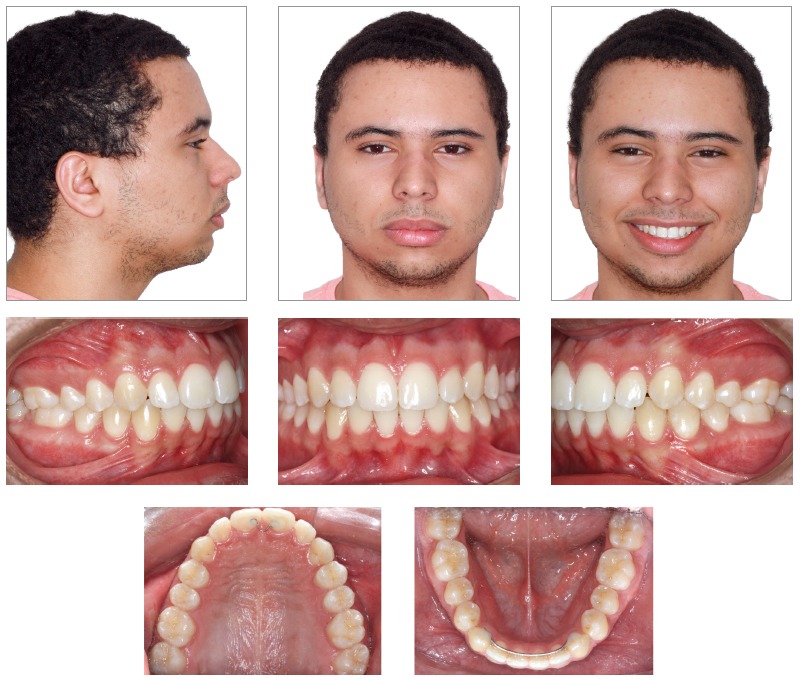
Follow-up intraoral and facial photographs 5 years after orthodontic treatment.

**Figure 15:**
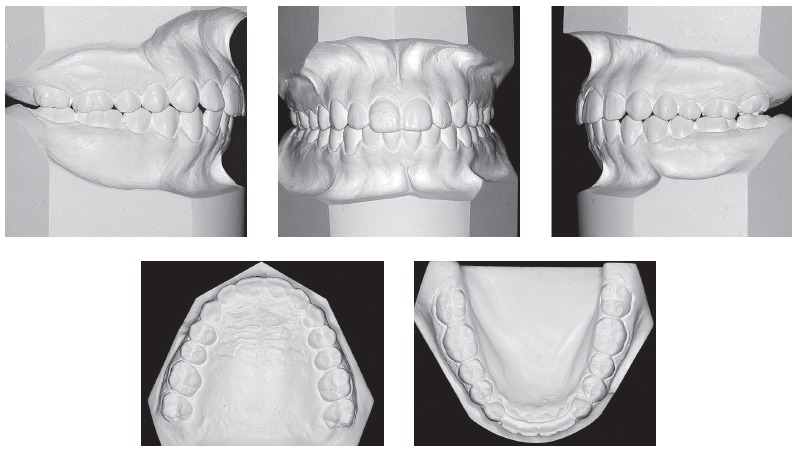
Follow-up models, 5 years after orthodontic treatment.

**Figure 16:**
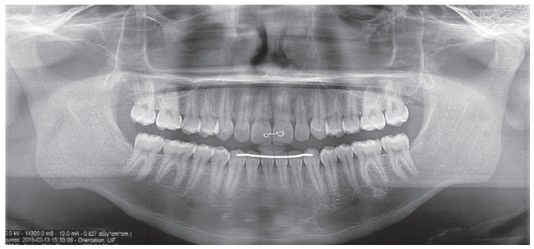
Follow-up panoramic radiograph, 5 years after orthodontic treatment.

**Figure 17:**
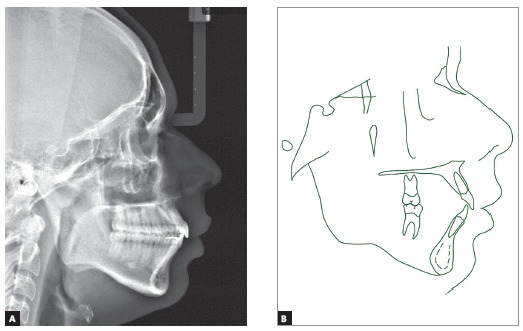
Follow-up profile cephalometric radiograph (A) and cephalometric tracing (B), 5 years after orthodontic treatment.

The five year follow-up after the end of the active treatment (Figs 14 to 17) showed stability of the obtained results from both occlusal and skeletal/facial perspectives. The functional aspect is still very well balanced. 

**Figure 18:**
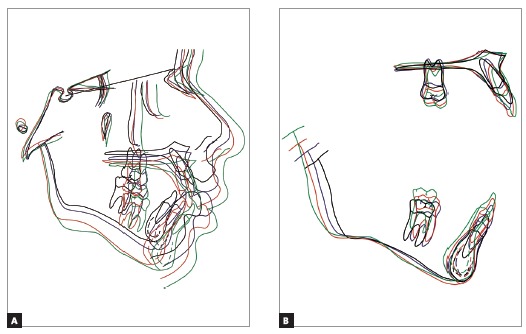
Total (A) and partial superimposition (B) of initial (black), intermediate (blue), final (red) and 5 years follow-up post-retention (green) cephalometric tracings.

Figure 18 illustrates the skeletal behavior throughout the treatment, by means of the cephalometric tracings superimposition. Vertical growth was predominant in the face, following the intrinsic facial growth pattern. Maxillary anterior displacement control was effective. Mandible achieved a good sagittal gain, specially after the use of the orthopedic appliance. During subsequent phases, a major inferior displacement was observed if compared to the anterior movement. Overbite and overjet were effectively corrected, besides the relevant improvement of the facial profile. 

Partial superimpositions revealed stable upper molars after the orthopedic phase, followed by extrusion and some mesial displacement along the subsequent phases. Upper incisors presented a marked uprighting immediately after orthopedics and extrusion in all phases. 

In the mandible, some extrusion was observed and slight mesial movement of the molars, besides extrusion and uprighting of the lower incisors, except during the retention phase, when a buccal movement was observed in these teeth. Mandible body and ramus observation revealed that an effective sagittal growth took place, with a good height gain along the ramus.

## FINAL REMARKS 

Treatment was based on the possibility of coupling skeletal correction to the orthopedic mandible advancement.^7^ ^-^ ^11^ Anterior open bite was related both to functional changes (thumb sucking, mouth breathing, atypical swallowing and altered speech) as to the clockwise rotation tendency presented by the mandible due to the strong vertical growth component - which by itself, was quite a limiting factor against more expressive sagittal gains.^12^ The age for intervention, despite a bit early from the pubertal growth spurt^13^ ^-^ ^14^ perspective, was adequate given the need for immediate action as to avoid the risk of trauma to anterior teeth and to improve patient’s self esteem, which had been deeply affected by the antisocial consequences of the malocclusion. 

Thus, with patient’s cooperation, the orthopedic advancement approach was shown to be a very efficient treatment strategy, providing the patient with a new functional-skeletal condition, achieved by an uneventful corrective phase. Positive aspects were also seen on perioral muscle tonus and function. Skeletal goals were met despite the limitations imposed by the facial pattern, presenting vertical growth and mandibular retrusion hereditary tendencies as additional hurdles. 

A functional and aesthetic occlusion was achieved with the effective correction of both overbite and overjet typical from the Class II division 1 malocclusion. The need for vertical Class II elastics was considered subtle, given the excellent orthopedic response, what allowed for root integrity preservation. Profile and smile were also very positively impacted. No speech therapy was prescribed, since the anatomical correction and functional stimuli of the first treatment phase were enough to trigger a functional spontaneous correction. Stability was observed throughout the five years of follow-up after the orthodontic therapy was finished.
